# Successful Ultra-Conservative Management of a Mandibular Premolar with Dens Invaginatus 

**DOI:** 10.22037/iej.v12i3.16559

**Published:** 2017

**Authors:** Ramin Abazarpour, Masoud Parirokh, Aida Farhadi, Zahra Jalali, Nasir Kheirabadi

**Affiliations:** a * Private Practice, Zahedan, Iran; *; b * Endodontology Research Center, Kerman University of Medical Sciences, Kerman, Iran; *; c * Endodontic Department, Dental School, Kerman University of Medical Sciences, Kerman, Iran; *; d * Restorative Department, Dental School, Zahedan University of Medical Sciences, Zahedan, Iran*

**Keywords:** Apical Plug, Calcium-enriched Mixture Cement, Cone-Beam Computed Tomography, Dens Invaginatus, Non-surgical Endodontic Treatment

## Abstract

Dens invaginatus is one of the most common anomalies of tooth structure. It is caused by the invagination of the crown surface during odontogenesis that enters the pulp chamber of the affected tooth. Depending on the complexity of invagination, the tooth might present with pulp necrosis, open apex and a complicated root canal system. This case report presents an Oehlers’ type 2 dens-invaginatus in a mandibular premolar with chronic apical abscess. In most cases, dens invaginatus is removed during treatment. However, in this case report, based on cone-beam computed tomography (CBCT) evaluation, non-surgical treatment and maintenance of the invaginated segment was chosen in order to prevent compromising the tooth structure and its susceptibility to future root fracture. This is a new treatment approach and has not been performed in previous reports. Calcium-enriched mixture (CEM) cement was used as an apical plug followed by gutta-percha in warm vertical compaction for root canal obturation. The case was followed up for 36 months after treatment. This report highlights the importance of selecting the appropriate treatment approach based on CBCT evaluation.

## Introduction

**Figure 1:**
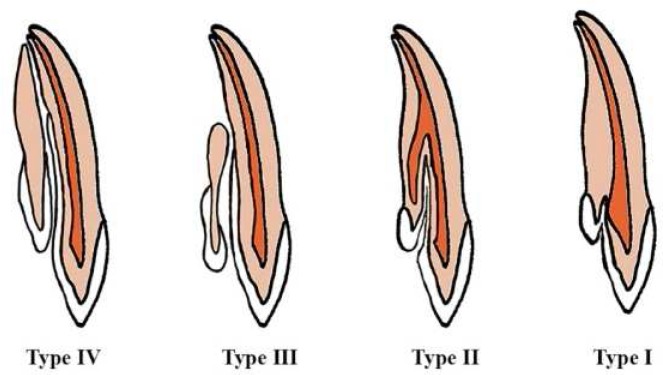
Classification of dens invaginatus by Oehlers

Dens invaginatus is a developmental condition affecting tooth structure prior to calcification of dentin matrix. This condition has also been termed as dens in dente, invaginated odontome, dilated gestant odontome, dentoid in dente and tooth inclusion [1, 2]. Several theories have been put forward for the etiology of dens invaginatus [3, 4]. These include the folding in of the enamel organ because of the adjacent tooth germ pressure and rapid formation of the internal enamel epithelium into the adjacent dental papilla during tooth development and presence of a small defect in the enamel epithelium that allows the adjacent epithelium to grow and develop continuously and absence of certain intercellular signal molecules causing dental anomalies and infection during tooth development [1-4]. Based on the review paper by Alani [1], genetic factor is the most likely cause of this anomaly. The prevalence of dens invaginatus in the general population has been reported from 0.04% to 10% [5]. Maxillary permanent lateral incisors are the most commonly affected teeth. However, any tooth, including supernumerary and primary teeth, could be affected. However, mandibular teeth are rarely affected [6]. Oehlers has classified dens invaginatus according to the extent of anomaly (Figure 1). Type I is defined as an enamel-lined invagination limited to the coronal part of the tooth. Type II is presented as a lesion extending beyond cemento-enamel junction and ending in a blind sac. Type III is the most complicated type in which invagination has penetrated the root laterally and an apical foramen separate from the main tooth apex is formed which complicates the process of endodontic treatment[1, 7, 8]. Thus far, the treatment used in case reports has mostly been through an orthograde approach and the removal of the invagination in Oehlers' class II cases. In this case report, a new treatment approach is introduced that was planned following cone-beam computed tomography (CBCT) examination of the tooth prior to treatment to determine the necessity of removing dens invaginatus during root canal treatment. Calcium-enriched mixture (CEM) cement apical plug and gutta-percha in warm vertical compaction technique were used in the treatment plan and the patient was followed up for 18, 24, and 36 months post treatment.

## Case Report

**Figure 2:**
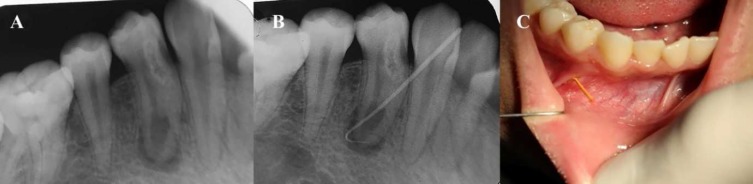
A) Preoperative periapical radiograph of the first mandibular premolar with chronic periapical pathosis; B) Periapical radiography of tracing of the sinus tract; C) Tracing of the sinus tract with #25 gutta-percha

**Figure 3:**
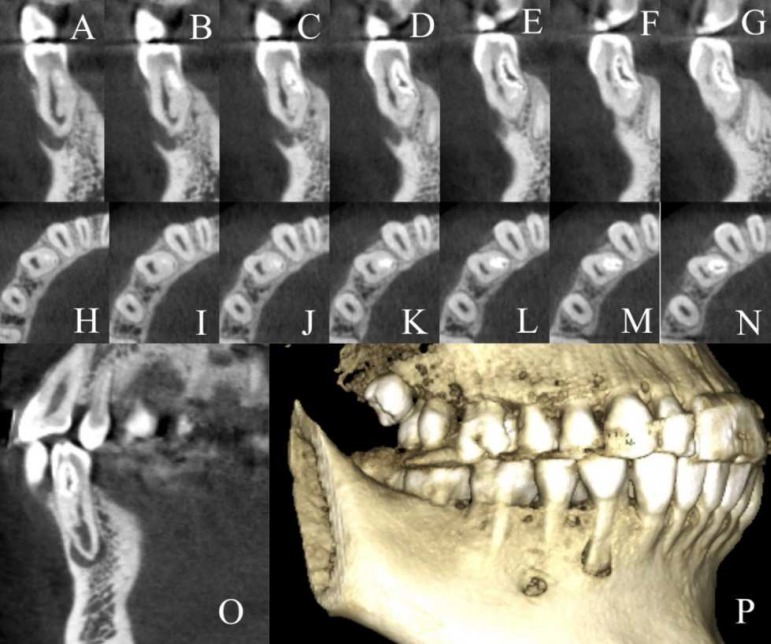
A to O) Confirmation of the extent of dens invaginatus and the size of periapical pathosis using cone-beam computed tomography images; P) Cone-beam computed tomography images ‎3D reconstruction (Buccal cortex perforation was observed adjacent to the affected tooth

A 19-year-old female patient was referred by a dentist to the Endodontic Department of the School of Dentistry, Kerman University of Medical Sciences, Kerman, Iran, with the chief complaint of infection and suppuration around the right anterior mandibular gingivae. The patient had noncontributory medical history. Intraoral clinical examinations revealed the involvement of tooth #28 and presence of an intraoral sinus tract. The tooth did not respond to electric pulp tester (Element Diagnostic Unit; SybronEndo, Glendora, CA, USA). The cold test was performed using a cold spray (Roeko Endo Frost; Roeko, Hangenav, Germany) which was sprayed on a cotton pellet and rapidly placed on the middle third of the buccal surface and hot stimulation was performed with warm gutta-percha (Ariadent, Tehran, Iran). However, the result of none of the tests was positive. The tooth was sensitive to the percussion test. The result of periodontal probing test with a Williams probe (Hu-Friedy Mfg. Co., LLC, UK) and mobility of the teeth were within normal ranges. A radiography was obtained and a radiolucent lesion was detected at the apical region of the tooth (Figure 2A). The sinus tract was traced with #25 gutta-percha and radiography was taken again (Figure 2B). In the tracing image, the gutta-percha entered the apical zone of tooth #28 (Figure 2C). The tooth was diagnosed with pulpal necrosis and chronic apical abscess. CBCT and Planmeca Romexis dental Software (2.3.1.R, PlanmecaOY, Helsinki, Finland) were used to assess the extent of the lesion (Figure 3A to P). The CBCT three-dimensional reconstruction revealed buccal cortex perforation adjacent to the affected tooth and type II dens invaginatus. 

**Figure 4.**
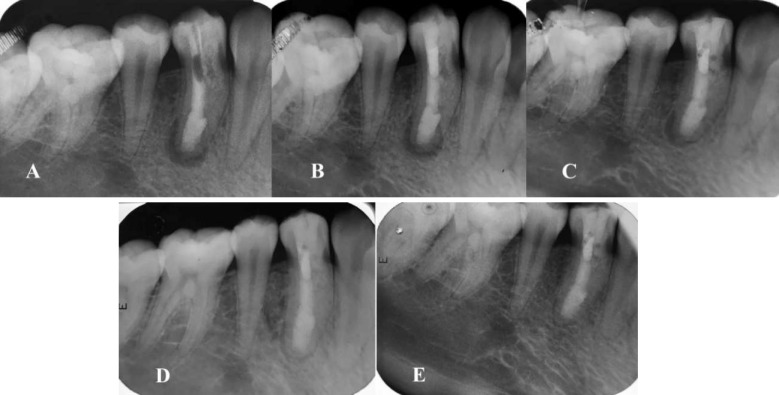
A) The ‎calcium enriched mixture ‎cement apical plug placement at the root canal and temporary restoration; B)‎ Calcium-enriched mixture ‎cement apical plug and coronal gutta-percha placement using vertical compaction technique; C) Follow-up radiograph of the tooth revealing a healing lucent lesion with diminished size 18 months after initial visit; D) Follow-up radiograph of the tooth revealing a healing lucent lesion with diminished size 24 months after initial visit; E) Periapical radiograph 36 months after the treatment

For the treatment procedure, non-surgical endodontic treatment with CEM cement (Bionque Dent; YektazistDandan Co., Tehran, Iran) without the removal of the dens invaginatus was chosen because CBCT evaluation showed no communication from the dens invaginatus with PDL. In addition, removing dens invaginatus may dramatically influence tooth structure. 

During the first appointment, using a side-loading cartridge aspirating syringe (Dena Instruments, Forgeman Instruments Co., Sialkot, Pakistan) and a 27-G 31-mm needle (Nik Rahnama Kar Co., Tehran, Iran), mental nerve block was conducted with 2% lidocaine containing 1:80000 epinephrine (Persocaine; DaruPakhsh, Tehran, Iran). The affected tooth was isolated with a rubber dam and access cavity preparation was performed with a diamond fissure bur (Diatech Dental, Heerbrugg, Switzerland). For root canal measurement, first, using #2 Gates Glidden drills (Mani, Tochigi, Japan), the coronal third of all root canals were expanded. Then, using a Root ZX apex locator (J. Morita Corporation, Kyoto, Japan) and #15 K-file (Mani, Tochigi, Japan), the root canal length was measured. The working length was confirmed through radiographic imaging. After confirmation of the working length, root canal instrumentation was completed using a stainless steel K-file of up to size #80 (Mani, Tochigi, Japan) through crown down instrumentation technique. The root canal space was irrigated with NaOCl (5.25%). Calcium hydroxide powder (Golchai, Tehran, Iran) was mixed with saline and was placed as intracanal medicament for a week and the tooth was temporarily restored with Cavisol (Golchai, Tehran, Iran). 

On the second visit, clinical examination revealed healing of the sinus tract. After local anesthesia and isolation with rubber dam, temporary restoration was removed. The calcium hydroxide dressing was removed with saline and alternating irrigation with 5.25% NaOCl and 17% ethylene diamine tetra acetic acid (EDTA) (Ariadent, Tehran, Iran) solutions. Subsequently, the canal was dried with paper points (Ariadent, Tehran, Iran). CEM cement powder and liquid were mixed according to the manufacturer's instructions. The mixture was inserted in to the dried canals using a sterile amalgam carrier. Then, it was gently adapted to the apical portion of the root canals using pre-fitted root canal pluggers (M-series, Dentsply Sirona Endodontics, Tulsa, OK, USA) to place an apical plug with the thickness of 3-4mm. Next, a moist cotton pellet was placed over the plug and again the tooth was restored temporarily. Correct placement and the CEM cement plug thickness was assessed radiographically (Figure 4A).

At the third and final visit, first, local anesthesia was administered and the tooth was isolated with a rubber dam. Next, temporary restoration was removed, and after irrigation, CEM cement setting was evaluated. Then, the root canal was filled with gutta-percha and AH-26 sealer (Dentsply Sirona Endodontics, Tulsa, OK, USA) using warm vertical compaction technique. The tooth was restored with resin composite (Z100, 3M ESPE, Premier, Norristown, PA, USA) and bonding (Single Bond, 3M ESPE, Premier, Norristown, PA, USA) as the final restoration (Figure 4B). In order to prevent future coronal leakage* via* dens invaginatus, a coronal cavity was prepared at the pit of the invaginatus and was restored with the composite resin.

The patient was followed up for 18, 24, and 36 months thereafter and the healing of the lesion was radiographically evaluated (Figure 4C, D and E). The lesion healed during the follow-up period.

## Discussion

In this case report, the successful conservative treatment of a premolar tooth with dens invaginatus without removing the anomaly has been presented. In most cases, dens invaginatus is accidentally discovered in radiographs, since, it usually develops in to pulpal necrosis without any significant symptoms. Thus, early diagnosis and treatment of dens invaginatus is necessary in order to prevent further pulp necrosis [9]. Different methods are used to treat dens invaginatus, such as fissure sealant and conservative restorations at early stages, root canal therapy, and surgical endodontic treatment. Recommended endodontic interventions for dens invaginatus are root canal treatment, apical surgery, intentional replantation, and extraction [10, 11]. The latter is only recommended when other treatment modalities have failed or supernumerary teeth are involved [12]. Most endodontists prefer nonsurgical approaches (orthograde) toward the treatment of dens invaginatus. In the present case, the orthograde treatment which was placement of CEM cement apical plug was chosen for treating the infected tooth.

Teeth with dens invaginatus are susceptible to caries and endodontic treatment is usually necessary for these teeth [13]. Teeth with dens invaginatus have many pits with defective or even absent enamel which lead to the colonization of microorganisms and further development of caries and pulp involvement which eventually necessitates endodontic treatment [14]. In this case report, in order to prevent future microorganism invasion through the anomaly, the coronal pit was restored with composite resin. 

CBCT can be a useful imaging technique. However, there are some concerns regarding the x-ray dosage. Both the American Association of Endodontists (AAE) and European Society of Endodontology (ESE) have recommended the use of CBCT for detection of complex root canal anatomy [15, 16]. Agrawal *et al. *[17] have noted that diagnostic and treatment planning can be improved through the use of CBCT imaging in suspected cases of dens invaginatus. They have also noted that it provides greater understanding of the complex radicular configurations of teeth with dens invaginatus [17]. However, before performing CBCT, the consideration of risk versus benefit should be taken into account [17]. In this case study, CBCT revealed the exact root canal anatomy, extent of the invagination, and size of the periradicular lesion. With the use of CBCT imaging, it was possible to determine suitable treatment plans and prevent complications.

The present case was a type II invagination based on Oehlers’ classification with no evident apical perforation of the sac, and it was not related to the apical pathosis. In the current case, CBCT imaging was used and it improved diagnostic and treatment planning capabilities for the anomaly as well as root canal configurations. Based on the image, non-surgical treatment without the removal or filling of the dens invaginatus was performed. In previously published case reports, the authors have discussed conservative protocols of dens invaginatus management using a minimally invasive nonsurgical treatment option; however, the invagination was removed or filled with root canal filling materials [17-20].

While performing root canal treatment, the removal of all debris from the root canal system and obturation of the root canal space with a biocompatible material is essential. Er *et al. *[12] successfully treated a mandibular premolar with type II dens invaginatus using gutta-percha and mineral trioxide aggregate (MTA) at the apical third of the tooth. Maintaining the invagination intact might result in some challenges in root canal instrumentation and shaping because of inaccessible areas on root canal walls. Therefore, 5.25% NaOCl solution was used for disinfection and intracanal irrigation because of its characteristics such as tissue dissolution capacity [21], smear layer removal potential during endodontic treatment [22] and effectiveness in killing *Enterococcus faecalis* after a 2-min contact time [23]. Calcium hydroxide was also used as intracanal dressing, because it is the most commonly used intracanal treatment in cases diagnosed with necrotic pulps and there is clinical evidence of its use in teeth with apical pathosis prior to definitive root canal filling [24].

Pace *et al. *[25] and Witherspoon *et al. *[26] reported high success rates in clinical and radiographic evaluation of MTA apical plug techniques in one or two visits. MTA is a kind of bioactive endodontic cement which is suitable for the artificial apical barrier technique because of its superior sealing ability and biocompatibility [27, 28]. However, some previous studies have shown that the sealing ability of white MTA apical plugs is adversely affected by calcium hydroxide use as intracanal medicament [29]. Nevertheless, in an *in vitro* study, it was reported that the insertion of calcium hydroxide between treatment sessions would not negatively affect the marginal adaptation of MTA or CEM cement as apical plug [30]. This finding was inconsistent with that of the present study on CEM cement.

In another study, it was reported that irrigation with NaOCl and EDTA, alternatively, resulted in complete calcium hydroxide removal from dentinal walls [31]. Thus, this method of irrigation was used on the second appointment to completely remove calcium hydroxide. Due to its previous successful applications as an artificial apical barrier, bioactive endodontic cement (CEM cement) was used in the current study as apical plug [32, 33]. The coronal part of the canal was filled with gutta-percha and restored with resin composite. 

## Conclusion

The present case reported a non-surgical root canal treatment with CEM cement as an apical plug without the removal of dens invaginatus. CBCT imaging was a useful aid for the practitioner assisting in understanding morphology and in decision making and treatment planning. 
